# Identification of immune-related hub genes and analysis of infiltrated immune cells of idiopathic pulmonary artery hypertension

**DOI:** 10.3389/fcvm.2023.1125063

**Published:** 2023-02-28

**Authors:** Yubin Chen, Tianyu Ouyang, Yue Yin, Cheng Fang, Can-e Tang, Longtan Jiang, Fanyan Luo

**Affiliations:** ^1^Department of Cardiac Surgery, Xiangya Hospital, Central South University, Changsha, Hunan, China; ^2^Department of Endocrinology, Xiangya Hospital, Central South University, Changsha, Hunan, China; ^3^The Institute of Medical Science Research, Xiangya Hospital, Central South University, Changsha, Hunan, China; ^4^National Clinical Research Center for Geriatric Disorders, Xiangya Hospital, Central South University, Changsha, Hunan, China

**Keywords:** idiopathic pulmonary artery hypertension, biomarker, immune gene, immune cells, inflammation, ceRNA network

## Abstract

**Objectives:**

Idiopathic pulmonary artery hypertension (IPAH) is a rare but life-threaten disease. However, the mechanism underlying IPAH is unclear. In this study, underlying mechanism, infiltration of immune cells, and immune-related hub genes of IPAH were analyzed via bioinformatics.

**Methods:**

GSE15197, GSE48149, GSE113439, and GSE117261 were merged as lung dataset. Weighted gene correlation network analysis (WGCNA) was used to construct the co-expression gene networks of IPAH. Gene Ontology and pathway enrichment analysis were performed using DAVID, gene set enrichment analysis (GSEA), and gene set variation analysis (GSVA). Infiltration of immune cells in lung samples was analyzed using CIBERSORT. GSE22356 and GSE33463 were merged as peripheral blood mononuclear cells (PBMCs) dataset. Immune-related differentially expressed genes (IRDEGs) of lung and PBMCs dataset were analyzed. Based on the intersection between two sets of IRDEGs, hub genes were screened using machine learning algorithms and validated by RT-qPCR. Finally, competing endogenous RNA (ceRNA) networks of hub genes were constructed.

**Results:**

The gray module was the most relevant module and genes in the module enriched in terms like inflammatory and immune responses. The results of GSEA and GSVA indicated that increasement in cytosolic calcium ion, and metabolism dysregulation play important roles in IPAH. The proportions of T cells CD4 memory resting and macrophage M1 were significantly greater in IPAH group, while the proportions of monocytes and neutrophils were significantly lower in IPAH group. IRDEGs of two datasets were analyzed and the intersection between two set of IRDEGs were identified as candidate hub genes. Predictive models for IPAH were constructed using data from PBMCs dataset with candidate hub genes as potential features via LASSO regression and XGBoost algorithm, respectively. *CXCL10* and *VIPR1* were identified as hub genes and ceRNA networks of *CXCL10* was constructed.

**Conclusion:**

Inflammatory response, increasement in cytosolic calcium ion, and metabolism dysregulation play important roles in IPAH. T cells CD4 memory resting and macrophage M1 were significantly infiltrated in lung samples from patients with IPAH. IRDEGs of lung dataset and PBMCs dataset were analyzed, and *CXCL10* and *VIPR1* were identified as hub genes.

## Introduction

Pulmonary artery hypertension (PAH) is a rare but life-threaten disease which is defined as a resting mean pulmonary arterial pressure of higher than 20 mmHg, pulmonary artery wedge pressure of lower than 15 mmHg, and an increasement in pulmonary vascular resistance of at least 3 Wood units (1). The prevalence of PAH ranges from 11 to 26 cases per million adults based on different registries (2). The incidence of PAH in women is four-fold higher than in men, but the prognosis of women with PAH is better than men with PAH (3, 4). In the last two decades, the development of treatment for PAH has been made, but the prognosis of PAH is still poor with one year survival rates varying from 86 to 90% (2) and the mean length of stay and inpatient mortality do not be significantly improved (5).

Variants in *bone morphogenetic protein receptor 2* (*BMPR2*) gene, specific drugs like methamphetamine, connective tissue disease, and congenital heart disease are important causes of PAH (6). The pathological changes of PAH include vascular remodeling, plexiform lesions, and fragmentation of the elastic lamina (7, 8). These changes would increase the pulmonary vascular resistance and eventually lead to the obliteration of small precapillary artery and arteriole (9). Idiopathic PAH (IPAH) is defined as hemodynamic changes of PAH which is not associated with other disease process (6). According to the REVEAL registry, there were 46% of 2525 patients with PAH had IPAH (10). And the REVEAL registry reported that the 5-years survival of patients with newly diagnosed IPAH was 68% (11). But the mechanisms underlying the development and progression of IPAH is largely unknown. And there are few biomarkers for IPAH and therapy targeting vascular remodeling. Hence, further exploration on mechanisms of IPAH is needed.

Inflammation plays an important role in IPAH and histologic studies revealed that lung tissues from patients with IPAH were infiltrated with immune cells including lymphocyte, macrophage, and mast cell (12). In addition, the concentrations of circulating inflammatory cytokines like interleukin (IL)-1β and tumor necrosis factor (TNF)-α were significantly increased in patients with IPAH (13). Peripheral blood mononuclear cells (PBMCs) consist of lymphocytes, monocytes and other cell types and is closely related to the immune response. There were studies suggested that PBMCs significantly associated with the development of IPAH (14, 15). Integrated bioinformatic analysis of PBMCs gene expression dataset of patients with IPAH might reveal new mechanisms of IPAH.

This study aimed to construct IPAH-related gene co-expression networks, conduct enrichment analysis, analyze the infiltration of immune cells in lung tissues from patients with IPAH, and analyze the immune-related differentially expressed genes (IRDEGs). Finally, the intersection between IRDEGs of lung tissue and PBMCs from patients with or without IPAH were taken and hub genes of IPAH were further identified from the intersection using machine learning algorithm.

## Materials and methods

### Data acquisition and processing

**FIGURE 1:**
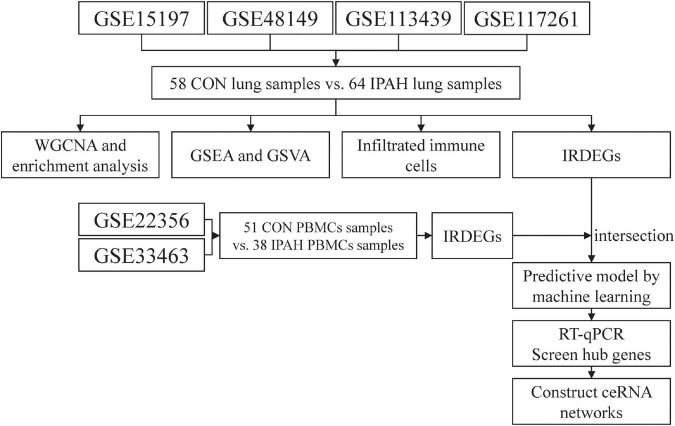
The flow chart of analysis in this study. CON, control; IPAH, idiopathic pulmonary artery hypertension; WGCNA, weighted gene correlation network analysis; GSEA, gene set enrichment analysis; GSVA, gene set variation analysis; IRDEGs, immune-related differentially expressed genes; RT-qPCR, real time quantitative polymerase chain reaction; ceRNA, competing endogenous RNA.

The flow chart of analysis in this study is shown in Figure 1. The raw data of GSE15197 (including 13 lung samples from patients without IPAH and 18 lung samples from patients with IPAH), GSE48149 (including nine lung samples from patients without IPAH and eight lung samples from patients with IPAH), GSE113439 (including 11 lung samples from patients without IPAH and six lung samples from patients with IPAH), and GSE117261 (including 25 lung samples from patients without IPAH and 32 lung samples from patients with IPAH) were downloaded from the Gene Expression Omnibus (GEO) database.^1^ GSE15197 was based on GPL6480 (Agilent-014850 Whole Human Genome Microarray 4 × 44K G4112F) and the expression matrix was extracted from raw data and normalized using “limma” package in R software (version 4.1.2; R Foundation for Statistical Computing, Vienna, Austria). GSE48149 was based on GPL16221 (Illumina HumanRef-8 v3.0 expression beadchip) and the expression matrix was obtained from raw data and normalized using “lumi” package in R software (version 4.1.2). GSE113439 and GSE117261 were based on GPL6244 (Affymetrix Human Gene 1.0 ST Array) and the expression matrixes were extracted from raw data and normalized using “oligo” package in R software (version 4.1.2). Thereafter, these expression matrixes were annotated using the “dplyr” and “limma” packages in R software (version 4.1.2). After that, the batch effect between these expression matrixes was removed using “sva” package in R software (version 4.1.2). Finally, these expression matrixes were merged as lung dataset (including 58 lung samples from patients without IPAH and 64 lung samples from patients with IPAH) for further analysis.

The raw data of GSE22356 (including ten PBMCs samples from patients without IPAH and eight PBMCs samples from patients with IPAH) and the normalized expression matrixes of GSE33463 (including 41 PBMCs samples from patients without IPAH and 30 PBMCs samples from patients with IPAH) were downloaded from the GEO database. GSE22356 was based on GPL570 (Affymetrix Human Genome U133 Plus 2.0 Array) and the expression matrix was obtained from raw data and normalized using “affy” package in R software (version 4.1.2). Then, two expression matrixes were annotated using the “dplyr” and “limma” packages in R software (version 4.1.2), and the batch effect between two expression matrixes was removed using “sva” package in R software (version 4.1.2). Finally, two expression matrixes were merged as PBMCs dataset (including 51 PBMCs samples from patients without IPAH and 38 PBMCs samples from patients with IPAH) for further analysis.

### Gene co-expression network construction by weighted gene correlation network analysis (WGCNA)

Gene co-expression networks based on lung dataset were constructed using the WGCNA package in R software (version 4.1.2) (16). Soft-thresholding power was used to construct a weighted adjacency matrix. Relationships between a single gene and others in the analysis were incorporated, and the adjacency matrix was transformed into the topological matrix (TOM). Then, a hierarchical clustering analysis of genes was performed using 1 – TOM as the distance measure. Thereafter, modules were detected using a dynamic tree cut algorithm with a minimum module size of 50 and a minimum cut height of 0.99. The correlation between each module and IPAH was calculated and shown in a heatmap. Finally, the most relevant gene module was selected for further analysis.

### Construction of protein-protein interaction (PPI) network

The gene module which was significantly related to IPAH with highest correlation coefficient was applied to construct PPI network using the Search Tool for the Retrieval of Interacting Genes (STRING) online tool^2^ and visualized using Cytoscape software (version 3.9.1; Institute for Systems Biology, Seattle, WA, USA). Then, cytoHubba, a plugin of Cytoscape software was used to identify the top ten key genes via the MCC method, and the corresponding PPI networks were constructed.

### Gene ontology (GO) and kyoto encyclopedia of genes and genomes (KEGG) pathway enrichment analysis of genes in the most relevant gene module

The Database for Annotation, Visualization and Integrated Discovery (DAVID, 2021 Update)^3^ were used to conduct GO and KEGG pathways enrichment analysis of genes in the most relevant gene module with the threshold of *p* value < 0.05.

### Gene set enrichment analysis (GSEA) and gene set variation analysis (GSVA)

The gene set files used in this study were downloaded from the Molecular Signatures Database (version: Human MSigDB v2022.1.Hs).^4^ The enrichment scores of GO and KEGG pathways terms in each group of lung dataset were calculated based on lung dataset using the GSEA software (version 4.2.3), and terms enriched in the IPAH group were identified. A nominal *p*-value (NOM *p*-val) of < 0.05 and false-discovery rate q value (FDR *q*-val) of <0.25 were considered as significantly enriched in the IPAH group.

GSVA was applied to evaluate GO and KEGG pathway terms enriched in each sample by converting the lung dataset into a gene set expression matrix using the “GSVA” package in R (version 4.1.2). After that, the differentially enriched terms between two groups were identified using R (version 4.1.2) with the threshold of p < 0.05. The differentially enriched terms were visualized using the “pheatmap” package in R (version 4.1.2).

### Analysis of infiltrated immune cells in lung samples

CIBERSORT^5^ is an algorithm that can analyze the relative abundance of 22 types of immune cells in each sample, including T-cells, B-cells, and macrophages. The parameters applied in this study were as follows: (I) 100 deconvolutions (Perm) and (II) *p* < 0.05. The analysis was based on lung dataset using “e1071” package in R software (version 4.1.2).

### Identification of differentially expressed genes (DEGs) and immune-related DEGs (IRDEGs) and prediction of transcription factors

The DEGs between two groups of lung dataset were identified using “limma” package in R (version 4.1.2) with the thresholds of *p* value < 0.05 and absolute value of log2 fold change (FC) > 0.3. The immune-related genes (IRGs) list was downloaded from Immport^6^. The IRDEGs were identified by taking the intersection between DEGs of lung dataset and IRGs list. The transcription factors of upregulated and downregulated IRDEGs were predicted using DAVID (2021 Update) with threshold of *p* value < 0.05. The interaction between IRDEGs and transcription factors was visualized using Cytoscape (version 3.9.1).

The DEGs between two groups of PBMCs dataset were identified using “limma” package in R (version 4.1.2) with the thresholds of *p*-value < 0.05 and absolute value of log2 fold change (FC) > 0.3. The IRDEGs of PBMCs dataset were identified as mentioned above.

### Construction of predictive model for IPAH and identification of hub gene for IPAH

The intersection between IRDEGs of lung dataset and IRDEGs of PBMCs dataset was taken and genes in the intersection were regarded as candidate hub genes. The predictive models for IPAH were constructed using least absolute shrinkage and selection operator (LASSO) regression and extreme gradient boosting (XGBoost) algorithm, respectively.

To construct predictive model for IPAH using LASSO regression, samples in PBMCs dataset were randomly divided into a training set (70%) and a testing set (30%). The predictive model was based on samples in the training set and candidate hub genes were selected as potential features in the model. “Glmnet” package in R (version 4.1.2) was applied to screen features and construct the model. The accuracy of the model was further validated using samples in testing set.

Similarly, samples in PBMCs dataset were randomly divided into a training set (70%) and a testing set (30%) before constructing model using XGBoost. “Xgboost” package in R (version 4.1.2) was applied to construct the model which was based on samples in the training set with candidate hub genes as features. In addition, feature importance of was analyzed and ranked.

The intersection between features in model constructed by LASSO regression and top 5 features in model constructed by XGBoost algorithm was taken, and genes in the intersection were identified as hub genes.

### Validation of hub genes

Blood samples were collected from patients with IPAH (*n* = 10) and patients without IPAH (*n* = 10) in Xiangya Hospital. The study was approved by the Ethics Committee of Xiangya Hospital, Central South University. Informed consent was obtained from all patients. PBMCs were separated from blood samples using Lymphocyte Separation Medium (40504ES60, Yeasen Biotechnology Co., Ltd., China) according to the manufacturer’s recommendation. Total RNA of PBMCs was extracted using RNAliquid Blood RNA Kit (RN2302, Aidlab Biotechnologies Co., Ltd., China) and reverse transcribed using the *Evo M-MLV* RT Mix Kit (AG11728, Accurate Biotechnology (Hunan)Co., Ltd., ChangSha, China) according to the manufacturer’s recommendations. Quantitative reverse transcription-polymerase chain reaction (RT-qPCR) was performed on a ViiA 7 system (Applied Biosystems) using All-in-One qPCR Mix (QP001, GeneCopoeia Inc.) for 40 cycles. GAPDH was used as a control for normalization. The primers used in this study are shown in Supplementary Table 1.

### Construction of competing endogenous RNA (ceRNA) networks of hub genes

The Encyclopedia of RNA Interactomes (ENCORI)^7^ database was used to construct ceRNA networks of hub genes (17). The micro-RNA (miRNA) which could interact with the hub genes was predicted using ENCORI with the threshold of number of supporting crosslinking-immunoprecipitation and high-throughput sequencing (CLIP-seq) experiments > = 2 and number of target-predicting programs > = 3. Then, the interaction pairs of miRNA-circRNA were predicted using ENCORI with the threshold of number of supporting CLIP-seq experiments > = 5 and supporting degradome-seq experiments > = 3. Finally, the circRNA-miRNA-mRNA interaction network was visualized using Cytoscape software (version 3.9.1).

### Statistical analysis

The relative expression levels of mRNA were presented as mean ± standard deviation (SD). The differences in proportions of immune cells and ratio of macrophage M1/M2 between different groups were analyzed by the Mann–Whitney *U* test. Pearson’s correlation analysis was used to analyze the relationship between different immune cells and the relationship between IRDEGs and proportions of immune cells. The diagnostic value of the predictive model was determined using receiver operating characteristic (ROC) curve and the area under ROC curve (AUC). Expression levels of mRNA in different group were compared using Student *t*-test. A value of *p* < 0.05 was considered to be statistically significant. Statistical analyses were performed using SPSS version 19 (IBM Corporation, Armonk, NY, USA) and R (version 4.1.2).

## Results

### Construction of IPAH-related gene co-expression networks and enrichment analysis of genes in the most relevant module

**FIGURE 2:**
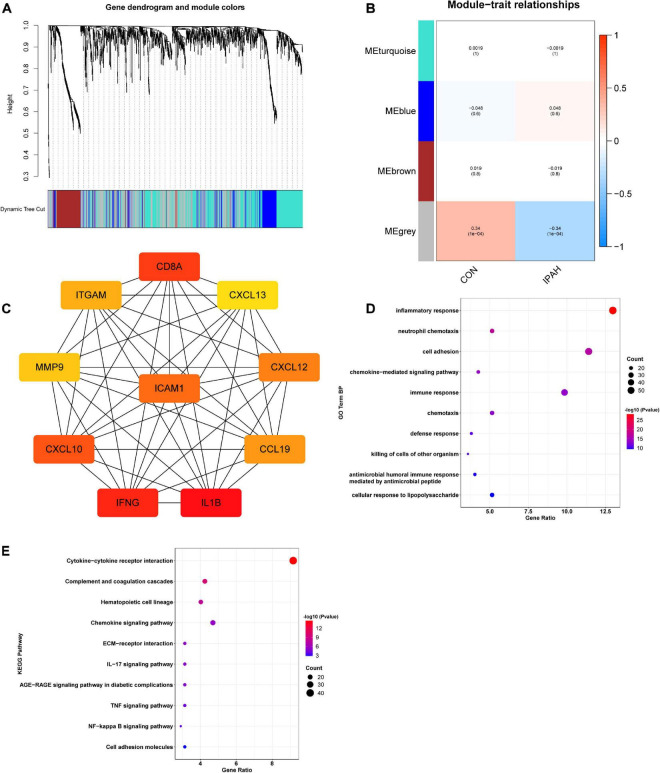
Construction of IPAH-related gene co-expression networks and enrichment analysis of genes in the most relevant module. **(A)** Dendrogram and clustering for identification of gene co-expression modules. **(B)** Correlation analysis of gene co-expression modules with IPAH. The numbers above brackets were correlation coefficients and the numbers in brackets were *p* values. **(C)** PPI network of key genes of the gray module. **(D,E)** GO BP and KEGG enrichment analysis of genes in the gray module. IPAH, idiopathic pulmonary artery hypertension; PPI, protein-protein interaction; GO, gene ontology; BP, biological process; KEGG, kyoto encyclopedia of genes and genomes.

WGCNA was applied to construct IPAH-related gene co-expression networks using the lung dataset. After analysis, four gene modules were obtained (Figure 2A). Then, the correlation between IPAH and each gene module was analyzed, and the results were displayed in Figure 2B. According to the absolute value of coefficient and *p*-value, the gray module was the most relevant module to IPAH. PPI network of the gray module was then constructed and the top ten key genes were identified using cytoHubba via MCC method. Top ten key genes included *IL1B*, *IFNG*, *CD8A*, *CXCL10*, *ICAM1*, *CXCL12*, *CCL19*, *ITGAM*, *MMP9*, and *CXCL13* (Figure 2C), most of which were inflammatory cytokines or chemokines. To further elucidate the functions of genes in the gray module, enrichment analysis was conducted. The results suggested that these genes were significantly enriched in terms of the GO biological process (BP) like inflammatory response, neutrophil chemotaxis, cell adhesion, chemokine-mediated signaling pathway, and immune response (Figure 2D). And the KEGG pathways in which these genes enriched were cytokine-cytokine receptor interaction, complement and coagulation cascades, hematopoietic cell lineage, chemokine signaling pathway, and extracellular matrix (ECM)-receptor interaction (Figure 2E).

### GSEA and GSVA results of lung dataset

Gene set enrichment analysis was conducted to further explore the terms of GO BP and KEGG pathways which enriched in IPAH group. As shown in Table 1, terms of GO BP included positive regulation of release of sequestered calcium ion into cytosol, positive regulation of glycolytic process, positive regulation of myoblast differentiation, positive regulation of cell adhesion mediated by integrin, and regulation of T cell chemotaxis were significantly enriched in IPAH group. Besides, KEGG pathways like ABC transporters, WNT signaling pathway, cell adhesion molecules, T cell receptor signaling pathway, and TGF beta signaling pathway were significantly enriched in IPAH group.

**TABLE 1:** The results of gene set enrichment analysis.

Gene set	NES	NOM *p*-val	FDR *q*-val
**GO term BP**			
Positive regulation of release of sequestered calcium ion into cytosol	2.12	<0.001	0.027
Regulation of release of sequestered calcium ion into cytosol	2.10	<0.001	0.020
Positive regulation of glycolytic process	2.00	<0.001	0.046
Calcium ion transmembrane import into cytosol	1.98	<0.001	0.047
Positive regulation of myoblast differentiation	1.95	<0.001	0.052
Integrin activation	1.93	0.004	0.058
Positive regulation of cell adhesion mediated by integrin	1.92	<0.001	0.056
Regulation of glucose import	1.89	<0.001	0.062
Regulation of T cell chemotaxis	1.86	0.002	0.069
T cell chemotaxis	1.82	0.002	0.088
**KEGG pathway**			
ABC transporters	1.91	<0.001	0.007
WNT signaling pathway	1.77	<0.001	0.021
Systemic lupus erythematosus	1.74	0.002	0.025
Intestinal immune network for IgA production	1.67	0.040	0.040
Cell adhesion molecules	1.60	<0.001	0.067
T cell receptor signaling pathway	1.58	0.002	0.076
TGF beta signaling pathway	1.54	0.009	0.094
Hedgehog signaling pathway	1.46	0.023	0.146
ECM receptor interaction	1.43	0.024	0.163
Chemokine signaling pathway	1.36	0.015	0.236

NES, normalized enrichment score; NOM *p*-val: nominal *p*-value; FDR *q*-val, false-discovery rate *q*-value; GO, gene ontology; BP, biological process; KEGG, kyoto encyclopedia of genes and genomes; ABC transporters, ATP binding cassette transporters; TGF, transforming growth factor; ECM, extracellular matrix.

**FIGURE 3:**
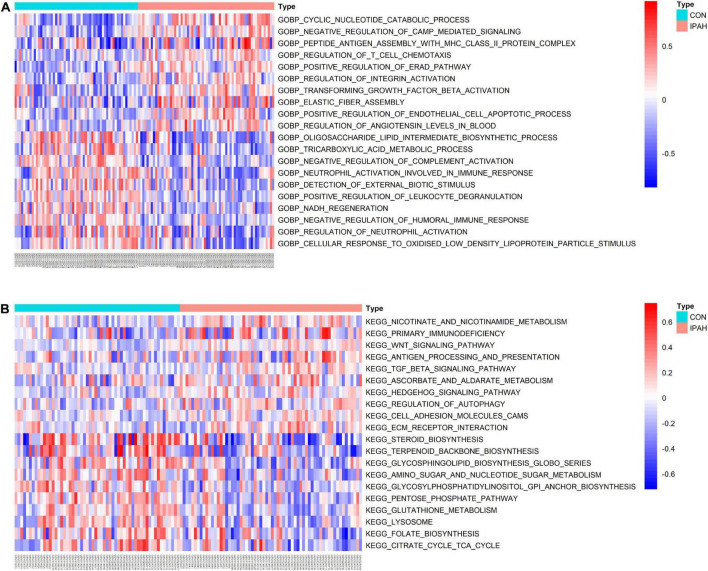
Results of GSVA. **(A)** Top 20 significantly different GO BP terms were shown in the heatmap. **(B)** Top 20 significantly different KEGG pathways were shown in the heatmap. GSVA, gene set variation analysis; GO, gene ontology; BP, biological process; KEGG, kyoto encyclopedia of genes and genomes.

GSVA was applied to analyze the differences in pathways between different groups and the differentially activated pathways were identified. Top 10 upregulated and downregulated terms of GO BP were shown in Figure 3A. The results indicated that negative regulation of CAMP mediated signaling, regulation of T cell chemotaxis, regulation of integrin activation, transforming growth factor beta activation, and positive regulation of endothelial cell apoptotic process were significantly activated in IPAH group, while oligosaccharide lipid intermediate biosynthetic process, tricarboxylic acid metabolic process, negative regulation of complement activation, neutrophil activation involved in immune response, and NADH regeneration were significantly downregulated in IPAH group. The heatmap of differentially activated KEGG pathways between CON and IPAH groups was displayed in Figure 3B. The results demonstrated that pathways like WNT signaling pathway, TGF beta signaling pathway, regulation of autophagy, cell adhesion molecules, and ECM receptor interaction were significantly upregulated in IPAH group, while terpenoid backbone biosynthesis, glycosphingolipid biosynthesis, amino sugar and nucleotide sugar metabolism, lysosome, and citrate cycle TCA cycle were significantly downregulated in IPAH group.

### Analysis of infiltrated immune cells in different lung samples

**FIGURE 4:**
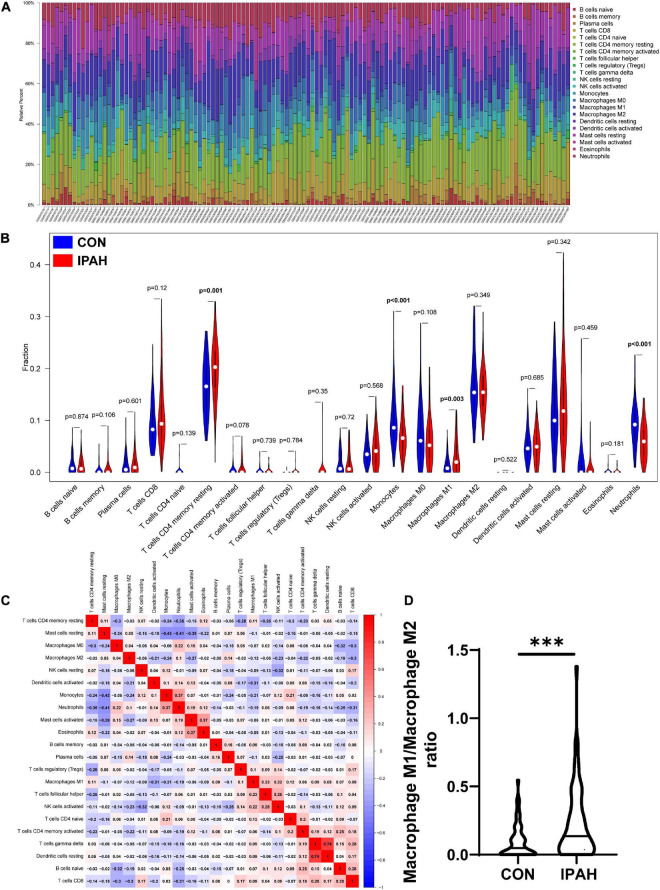
Analysis of infiltrated immune cells in different lung samples. **(A)** The relative percent of 22 kinds of immune cells in different lung samples. **(B)** The difference in proportions of immune cells in different lung samples. **(C)** The correlation between different immune cells. **(D)** The macrophage M1/macrophage M2 ratio in CON and IPAH groups. ^***^p < 0.001.

The infiltrated immune cells in different samples were analyzed using CIBERSORT and the overall relative abundances of 22 types of immune cells were shown in Figure 4A. Then, the difference in proportions of immune cells between CON and IPAH groups was analyzed and the results revealed that the proportions of T cells CD4 memory resting and macrophage M1 were significantly greater in IPAH group, while the proportions of monocytes and neutrophils were significantly lower in IPAH group (Figure 4B). The correlation between 22 kinds of immune cells was further analyzed and shown in Figure 4C. Finally, the macrophage M1/macrophage M2 ratio of different samples was calculated and the result indicated that the ratio was significantly higher in IPAH group (Figure 4D), which suggested severe inflammatory state in patients with IPAH.

### Identification of IRDEGs of the lung dataset and prediction of transcription factors of IRDEGs

**FIGURE 5:**
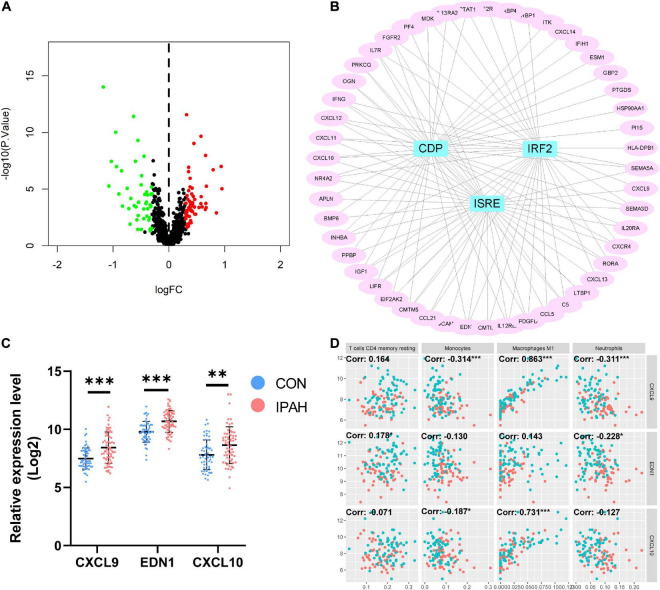
Identification of IRDEGs of the lung dataset and prediction of transcription factors of IRDEGs. **(A)** The volcano plot of IRDEGs of lung dataset. **(B)** PPI network of upregulated IRDEGs and the predicted transcription factors. **(C)** The relative expression levels of top three upregulated IRDEGs. **(D)** The correlation between top three upregulated IRDEGs and significantly changed immune cells. Dots in red: lung samples from patients without IPAH, dots in bule: lung samples from patients with IPAH. IRDEGs, immune-related differentially expressed genes; PPI, protein-protein interaction; IPAH, idiopathic pulmonary artery hypertension. ^**^p < 0.01, ^***^p < 0.001.

With the thresholds of *p*-value < 0.05 and absolute value of log2 FC > 0.3, there were 103 IRDEGs in lung dataset, including 56 upregulated IRDEGs and 47 downregulated IRDEGs (Figure 5A). Transcription factors of IRDEGs were predicted using DAVID and the results demonstrated that *CDP*, *IRF2*, and *ISRE* were the most relevant transcription factors of upregulated IRDEGs (Figure 5B). But for downregulated IRDEGs, there was no transcription factor that met the threshold of *p* value < 0.05. Top three upregulated IRDEGs were *CXCL9*, *EDN1*, and *CXCL10*, and expression levels of these genes were shown in Figure 5C. The correlation between top three upregulated IRDEGs and significantly changed immune cells was analyzed and results indicated that expression levels of *CXCL9* and *CXCL10* were negatively associated with the proportion of monocytes while positively associated with the proportion of macrophages M1 (Figure 5D).

### Analysis of IRDEGs of the PBMCs dataset and identification of candidate hub genes

**FIGURE 6:**
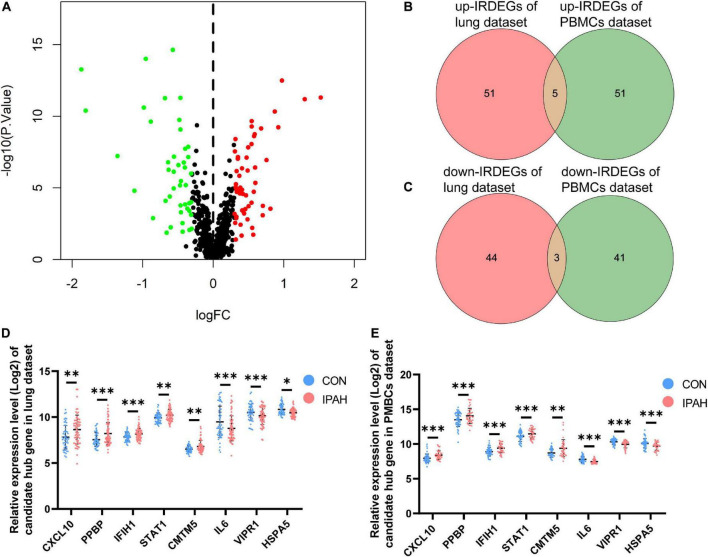
Analysis of IRDEGs of the PBMCs dataset and identification of candidate hub genes. **(A)** The volcano plot of IRDEGs of PBMCs dataset. **(B,C)** Venn plots of IRDEGs of lung and PBMCs datasets. **(D,E)** The relative expression levels of candidate hub genes in lung and PBMCs datasets, respectively. IRDEGs, immune-related differentially expressed genes; PBMCs, peripheral blood mononuclear cells. *p < 0.05, ^**^p < 0.01, ^***^p < 0.001.

Using the thresholds mentioned above, there were 100 IRDEGs in PBMCs dataset, including 56 upregulated IRDEGs and 44 downregulated IRDEGs (Figure 6A). Then, the intersection between IRDEGs of the lung dataset and PBMCs dataset was taken (Figures 6B, C). The intersection contained eight genes, five of which were upregulated genes including *CXCL10*, *PPBP*, *IFIH1*, *STAT1*, and *CMTM5*, and three of which were downregulated genes including *IL6*, *VIPR1*, and *HSPA5*. These genes were identified as candidate hub genes and expression levels of candidate hub genes in different datasets were shown in Figures 6D, E.

### Construction of predictive models, screening of hub genes, and construction of ceRNA networks of hub genes

**FIGURE 7:**
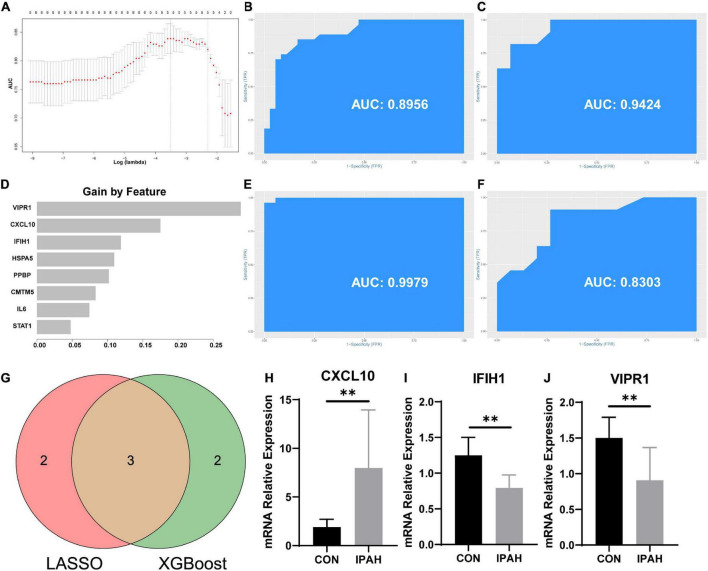
Construction of predictive models and screening of hub genes. **(A)** Select optimal lambda value for predictive model. **(B,C)** The ROC curves and AUC values of the predictive model constructed by LASSO regression in the training and testing sets, respectively. **(D)** The rank of feature importance in the predictive model constructed by XGBoost. **(E,F)** The ROC curves and AUC values of the predictive model constructed by XGBoost in the training and testing sets, respectively. **(G)** Venn plot of screening hub genes. **(H–J)** Expression levels of CXCL10, IFIH1, and VIPR1 in PBMCs samples. ROC curve, receiver operating characteristic curve; AUC, area under ROC curve; LASSO, least absolute shrinkage and selection operator; XGBoost, extreme gradient boosting. ^**^p < 0.01.

**FIGURE 8:**
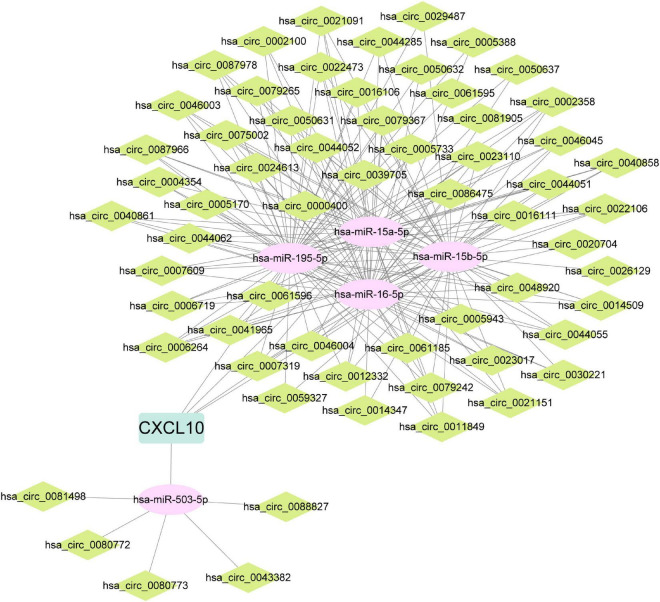
ceRNA network of CXCL10. ceRNA, competing endogenous RNA; CXCL10, C-X-C motif chemokine ligand 10.

Predictive models were constructed based on data from the PBMCs dataset with candidate hub genes as potential features. First, LASSO regression was applied to construct the predictive model. According to LASSO regression, *CXCL10*, *IFIH1*, *CMTM5*, *IL6*, and *VIPR1* were selected as features in the model, and the values of AUC of the model in training and testing sets were 0.8956 and 0.9424, respectively (Figures 7A–C). Then, XGBoost algorithm was utilized to construct the predictive model and all eight candidate hub genes were included in the model as features. The importance of features in the model was calculated and ranked (Figure 7D). The values of AUC of the model in training and testing sets were 0.9979 and 0.8303, respectively (Figures 7E, F). The intersection between features in model constructed by LASSO regression and top five features in model constructed by XGBoost algorithm was taken (Figure 7G). Genes in the intersection included *CXCL10*, *IFIH1*, and *VIPR1*. To further screen hub genes, mRNA expression levels of these genes in PBMCs samples from patients with or without IPAH were determined using RT-qPCR (Figures 7H-J). And the results suggested that the mRNA expression level of *CXCL10* was significantly increased in PBMCs samples from patients with IPAH, while the mRNA expression level of *VIPR1* was significantly decreased in PBMCs samples from patients with IPAH. In addition, the mRNA expression level of *IFIH1* was significantly decreased in PBMCs samples from patients with IPAH, which was not consistent with the result of IRDEGs. Therefore, *CXCL10* and *VIPR1* were identified as hub genes. Considering the advantages of miRNA and circRNA as biomarkers, ceRNA networks of hub genes were then constructed. The ceRNA network of *CXCL10* was constructed successfully and shown in Figure 8. But for *VIPR1*, there was no interaction pair of miRNA-mRNA according to the threshold of prediction.

## Discussion

PAH is an uncommon but devastating disorder which is characterized by the increasement in pulmonary vascular resistance (6). According to a French registry (*n* = 674), the incidence of PAH was about 2.4 cases per 1 million per year (18). Besides, the REVEAL registry (*n* = 2525) reported that the incidence of PAH was approximately 2.0 cases per 1 million per year (10). Most of symptoms of PAH are non-specific like dyspnea and fatigue, which affects the early diagnosis of PAH (19). In the last two decades, the treatments for PAH have developed rapidly. But these treatments mainly focus on relief symptoms, and currently there is no therapy directly target pulmonary vascular remodeling (20). Therefore, the prognosis of PAH is still poor and the five-year survival of patient with PAH ranged from 20.8% to 61% in different studies (2). IPAH is an important subgroup of PAH which meet hemodynamic changes of PAH but is not associated with other disease process (6). The proportion of IPAH in patients with PAH was 46% in REVEAL registry (10) and 39% in French registry (18). According to the REVEAL registry, the five-year survival of patients with IPAH was about 68% (11). It is hard to diagnose IPAH at early stage and there is no specific therapy for IPAH. Therefore, further exploration of potential mechanisms underlying IPAH is needed, which might provide novel biomarkers and therapeutic targets for IPAH.

In the first part of this study, GSE15197, GSE48149, GSE113439, and GSE117261 were merged as the lung dataset to explore the potential underlying mechanisms underlying IPAH. The lung dataset contained 122 lung samples, including 58 lung samples from patients without IPAH and 64 lung samples from patients with IPAH. The sample size of this study is greater than the bioinformatics researches of Zhao et al. (21), Qiu et al. (22), and Zeng et al. (23). To ensure the accuracy of the bioinformatics analysis, the batch effect between these datasets was removed before merging. To find out the potential mechanisms, IPAH-related gene co-expression networks were constructed using the lung dataset and the gray module was identified as the most relevant module. The PPI network of the gray module was constructed and key genes were screened. Key genes, and significantly enriched GO BP terms and KEGG pathways suggested that inflammation and immune response contributed to the development and progression of IPAH. These findings were consistent to the current opinion on IPAH. In the lung samples from patients with PAH, researchers have found the aggregate of small lymphoid and the highly organized lymphoid follicles (20). The neutrophil-to-lymphocyte ratio was increased in patients with PAH and the increasement in neutrophil-to-lymphocyte ratio was related to a poor clinical prognosis of patients with PAH (24, 25). The circulating concentrations of inflammatory cytokines were increased in patients with IPAH (13). In addition, histological studies have revealed that lung samples from patients with IPAH were infiltrated with lymphocyte, macrophage, dendritic cell, and mast cell (12, 26, 27), which reflected the severe inflammatory state in the lung. Not only immune cells, but also pulmonary arterial smooth muscle cell (PASMC), pulmonary arterial endothelial cell (PAEC), and fibroblast from patients with PAH exhibited proinflammatory phenotype (20). Inflammatory cytokines, chemokines and adhesion molecules like intercellular adhesion molecule (ICAM)1 were upregulated in these cell types and exacerbated the inflammatory response (20, 28, 29). However, treatment with steroid or aspirin could not ameliorate the pulmonary vascular remodeling in patients with IPAH (20). The fact suggested that more researches focusing on the relationship between inflammation and IPAH are needed to develop novel therapeutic strategy.

To further find out potential pathophysiological processes involved in the development and progression of IPAH, GSEA, and GSVA were conducted and the results indicated that regulation of release of sequestered calcium ion into cytosol and regulation of glycolytic process were significantly activated in patients with IPAH. The concentration of cytosolic calcium ion in PASMC is closely associated with the cellular function, and previous researches reported that the concentration of cytosolic calcium ion in PASMC from patients with PAH was significantly increased, which was partly mediated by activation of transient receptor potential channel (TrpC)6 and downregulation of voltage gated potassium channels like Kv1.5 (2, 30, 31). Increasement in cytosolic calcium ion could promote the contractility of PASMC, activate the proliferation by driving PASMC into cell cycles, and inhibit the apoptosis process, both of which eventually lead to the hyperplasia of PASMC and accelerate the process of vascular remodeling (30, 32). The metabolism pattern of cells in lung from patients with PAH is significantly changed and researches have indicated that glucose metabolism of PAEC and PASMC from patients with PAH is shifted from complete mitochondrial oxidative phosphorylation toward cytoplasmic glycolysis to pyruvate and ultimately lactate (33). The metabolism pattern is associated with increasement in glucose influx and is independent of oxygen availability (33, 34). The metabolic shift supports rapid proliferation of fibroblast and PAEC and avoiding mitochondrial apoptosis (35, 36). Based on the special metabolism pattern, specific therapeutic strategies have been developed like inhibition of pyruvate dehydrogenase kinase (PDK), which could suppress the mitochondrial oxidative phosphorylation (20). But effect of these treatment needs more clinical evidence.

Considering the important role of inflammation in IPAH, the infiltration of immune cells in lung samples from patients with IPAH was analyzed. The results revealed that the proportion of T cells CD4 memory resting was significantly greater in IPAH group. The finding is consistent with Mansueto et al., who reported that the CD4 positive T cells were significantly infiltrated in vascular lesions of patients with PAH (37). We also found that the proportion of macrophage M1 and the macrophage M1/macrophage M2 ratio was significantly greater in IPAH group. These results suggested a severe inflammatory response in patients with IPAH (38). Surprisingly, the results revealed that the proportion of neutrophils was significantly decreased in IPAH group. The role of neutrophils in PAH is still controversial. Li et al. reported that circulating count of neutrophil was positive associated with the pulmonary vascular resistance of patients with IPAH (39), while He et al. found that the circulating count of neutrophil was similar between patients with or without PAH (40). And there are few studies focusing on the effect of infiltrated neutrophil on IPAH, which needs further exploration.

As mentioned above, there is a lack of biomarker and therapeutic target for IPAH. Therefore, in the second part of this study, we aimed to screen hub genes, which might be biomarker or therapeutic target for IPAH. PBMCs are closely associated with IPAH (15). Immune and inflammation responses are two main pathophysiological processes involved in the development and progression of IPAH (41). So, genes in intersection between IRDEGs of the lung dataset and the PBMCs dataset were regarded as candidate hub genes. In these genes, we surprisingly found that the expression level of IL6 was significantly decreased in IPAH group of lung and PBMCs datasets. However, other researchers reported that the circulating concentration of IL6 was significantly increased in patients with PAH (13, 42). We speculated that the mRNA expression level and the circulating protein level might be different. And the regulation of transcription, translation, and secretion of IL6 in patients with IPAH needs further research. These genes were further screened using LASSO regression and XGBoost algorithm. Genes in intersection between features in model constructed by LASSO regression and top five features in model constructed by XGBoost algorithm were further validated using RT-qPCR. After that, *CXCL10* and *VIPR1* were identified as hub genes. CXCL10 (C-X-C motif chemokine ligand 10) is an important chemokine, which is upregulated in response to proinflammatory cytokines like TNF-α (43). Cunningham et al. reported that treatment with CXCL10 could trigger the apoptosis of PAEC, and inhibition of CXCL10 in rat could significantly reduce the severity of pulmonary hypertension (44). There are other bioinformatics researches identified CXCL10 as hub gene of IPAH (45, 46). VIPR1 (vasoactive intestinal peptide receptor 1) plays an important role in smooth muscle relaxation and acts as an anti-inflammatory cytokine (47), which is significantly downregulated in the IPAH group of lung dataset and PBMCs dataset. However, there is no research about the effect of VIPR1 on IPAH. In this study, hub genes were screened based on lung and PBMCs datasets, which meant hub genes could be detected in peripheral PBMCs and act as potential biomarkers. Hub genes were further validated using RT-qPCR in PBMCs samples. PBMCs samples are easier to obtain than lung tissue samples, which might facilitate the early diagnosis of IPAH. On the other hand, the dysregulation of hub genes in lung tissues and PBMCs samples emphasized the crucial roles of hub genes in the development and progression of IPAH. Therapy based on these genes might be a new area of IPAH study. miRNA and circRNA involve in the development and progression of IPAH, and are suitable to be biomarkers for IPAH (2, 48). Therefore, ceRNA networks of these hub genes were constructed. The ceRNA network of CXCL10 was constructed successfully. But for VIPR1, there was no interaction pair of miRNA-mRNA according to the threshold of prediction. The miRNA and circRNA in the ceRNA network might be potential biomarkers for IPAH but the diagnosis values of miRNA and circRNA need further validation.

Comparing with the bioinformatics analysis conducted by He et al. (46), this study included more samples in lung dataset and PBMCs dataset, which ensured the accuracy of this study. And before merging different gene expression datasets, batch effects were removed using “sva” package in R software (version 4.1.2). In addition to DAVID, GSEA and GSVA were applied to analyze the GO terms and KEGG pathways enriched in IPAH group. Besides, the infiltration of immune cells in lung tissues from patients with IPAH was investigated by CIBERSORT. LASSO regression and XGBoost algorithm were utilized to screen hub genes based on the lung and PBMCs datasets, and hub genes were further validated in PBMCs samples. The difference in hub genes between this study and study of He et al. (46) might be due to the difference in the selection of gene expression datasets and the way to screen hub genes. The bioinformatics study of Huang et al. selected GSE15197, GSE113439, GSE48149, and GSE33463 for analysis, which was similar with this study, but only GSE15197 was utilized to screen hub genes using LASSO regression and PPI network (49). In this study, GSE15197, GSE48149, GSE113439, and GSE117261 were merged as the lung dataset, and GSE22356 and GSE33463 were merged as the PBMCs dataset. The screening of hub genes was based on lung and PBMCs datasets using LASSO regression and XGBoost algorithm and hub genes were further validated in PBMCs samples. The difference in selection of gene expression dataset and the screening methods might explain the discrepancy of hub genes. Similarly, the difference in the results of infiltration of immune cells might be due to the difference in the sample size and the selection of gene expression dataset.

In conclusion, inflammatory response, increasement in cytosolic calcium ion, and metabolism dysregulation play important roles in IPAH. T cells CD4 memory resting and macrophage M1 were significantly infiltrated in lung samples from patients with IPAH. IRDEGs of lung dataset and PBMCs dataset were analyzed, and *CXCL10* and *VIPR1* were identified as hub genes.

## Data availability statement

The dataset used in this study can be found at: https://www.ncbi.nlm.nih.gov/geo/, accession numbers GSE15197, GSE48149, GSE113439, GSE117261, GSE22356, and GSE33463.

## Author contributions

YC analyzed the data, conducted experiments, completed figures, and wrote manuscript. TO, YY, and CF processed the raw data and completed the ceRNA network. C-eT, LJ, and FL designed the research. LJ and FL reviewed and edited the manuscript. All authors contributed to the article and approved the submitted version.

## Footnotes

1
https://www.ncbi.nlm.nih.gov/gds

2
https://cn.string-db.org/

3
https://david.ncifcrf.gov/home.jsp

4
http://www.gsea-msigdb.org/gsea/msigdb/index.jsp

5
https://cibersort.stanford.edu/

6
https://www.immport.org/home

7
https://starbase.sysu.edu.cn/
