# Supplementary material for: Identification of immune-related hub genes and analysis of infiltrated immune cells of idiopathic pulmonary artery hypertension
Source: Front Cardiovasc Med. 2023 Feb 28;10:1125063. doi: 10.3389/fcvm.2023.1125063 (PMC10011155; doi:10.3389/fcvm.2023.1125063)
Supplement: Supplementary file 1 [file Table_1.DOCX]

| GAPDH | Forward | 5’ CCAGCAAGAGCACAAGAGGAAGAG 3’ |
| --- | --- | --- |
|  | Reverse | 5’ GGTCTACATGGCAACTGTGAGGAG 3’ |
| CXCL10 | Forward | 5’ GTGGCATTCAAGGAGTACCTC 3’ |
|  | Reverse | 5’ TGATGGCCTTCGATTCTGGATT 3’ |
| IFIH1 | Forward | 5’ TCGAATGGGTATTCCACAGACG 3’ |
|  | Reverse | 5’ GTGGCGACTGTCCTCTGAA 3’ |
| VIPR1 | Forward | 5’ TCATCCGAATCCTGCTTCAGA 3’ |
|  | Reverse | 5’ AGGCGAACATGATGTAGTGTACT 3’ |

Supplementary Table 1: Primers used in this study
